# Advanced Skin Antisepsis: Application of UVA-Cleavable Hydroxyethyl Starch Nanocapsules for Improved Eradication of Hair Follicle-Associated Microorganisms

**DOI:** 10.3390/pharmaceutics15020609

**Published:** 2023-02-11

**Authors:** Loris Busch, Anna Maria Hanuschik, Yuri Avlasevich, Katrin Darm, Elisa F. Hochheiser, Christian Kohler, Evgeny A. Idelevich, Karsten Becker, Peter Rotsch, Katharina Landfester, Maxim E. Darvin, Martina C. Meinke, Cornelia M. Keck, Axel Kramer, Paula Zwicker

**Affiliations:** 1 Center of Experimental and Applied Cutaneous Physiology, Department of Dermatology, Venereology and Allergology, Charité—Universitätsmedizin Berlin, Corporate Member of Freie Universität Berlin and Humboldt-Universität zu Berlin, Charitéplatz 1, 10117 Berlin, Germany; 2 Department of Pharmaceutics and Biopharmaceutics, Philipps University Marburg, Robert-Koch-Str. 4, 35037 Marburg, Germany; 3 Institute of Hygiene and Environmental Medicine, University Medicine Greifswald, Ferdinand-Sauerbruch-Str., 17475 Greifswald, Germany; 4 Max Planck Institute for Polymer Research, Ackermannweg 10, 55128 Mainz, Germany; 5 Friedrich Loeffler—Institute of Medical Microbiology, University Medicine Greifswald, Ferdinand-Sauerbruch-Str., 17475 Greifswald, Germany; 6 Institute of Medical Microbiology, University Hospital Münster, Domagkstraße 10, 48149 Münster, Germany; 7 OSA Opto Light GmbH, Köpenicker Str. 325, 12555 Berlin, Germany

**Keywords:** hydroxyethyl starch, UVA-responsive, nanocapsules, SR101, hair follicles, cytotoxicity, antiseptics, dynamic light scattering, confocal laser scanning microscopy, UVA-LED

## Abstract

Hair follicles constitute important drug delivery targets for skin antisepsis since they contain ≈25% of the skin microbiome. Nanoparticles are known to penetrate deeply into hair follicles. By massaging the skin, the follicular penetration process is enhanced based on a ratchet effect. Subsequently, an intrafollicular drug release can be initiated by various trigger mechanisms. Here, we present novel ultraviolet A (UVA)-responsive nanocapsules (NCs) with a size between 400 and 600 nm containing hydroxyethyl starch (HES) functionalized by an *o*-nitrobenzyl linker. A phase transfer into phosphate-buffered saline (PBS) and ethanol was carried out, during which an aggregation of the particles was observed by means of dynamic light scattering (DLS). The highest stabilization for the target medium ethanol as well as UVA-dependent release of ethanol from the HES-NCs was achieved by adding 0.1% betaine monohydrate. Furthermore, sufficient cytocompatibility of the HES-NCs was demonstrated. On ex vivo porcine ear skin, a strong UVA-induced release of the model drug sulforhodamine 101 (SR101) could be demonstrated after application of the NCs in cyclohexane using laser scanning microscopy. In a final experiment, a microbial reduction comparable to that of an ethanol control was demonstrated on ex vivo porcine ear skin using a novel UVA-LED lamp for triggering the release of ethanol from HES-NCs. Our study provides first indications that an advanced skin antisepsis based on the eradication of intrafollicular microorganisms could be achieved by the topical application of UVA-responsive NCs.

## 1. Introduction

Mammalian body surfaces, including skin, represent a remarkably complex and highly variable microbiological environment [1,2,3]. The hair follicle constitutes a special habitat with a considerable bacterial reservoir comprising about 25% of all cultivated bacteria found on the skin [4]. With conventional skin antisepsis, a penetration into the deeper parts of the hair follicle is not possible which makes these parts a protected reservoir for microorganisms [5]. Being in the hair follicles, microorganisms are constantly released to the surface, leading to recontamination of the skin which can result in the development of surgical site infections (SSI). For the prevention of SSI, which are caused by endogenous pathogens in about 90% of all cases [6] and mainly by microorganisms in the depth of the skin surrounding the surgical field, an advanced skin antisepsis is necessary including an efficient targeting of hair follicles to prevent an intraoperative recolonization on the skin surface. 

The pathogen spectrum causing SSI varies depending on the location of the surgery field. In general surgery, the etiology of SSI has not significantly changed over the last 30 years. Gram-positive bacteria, e.g., coagulase-negative staphylococci (CoNS), *Staphylococcus aureus* and *Enterococcus* spp. as well as gram-negative bacteria, e.g., *Escherichia coli*, *Enterobacter* spp., *Klebsiella* spp. and *Pseudomonas aeruginosa*, are the most common findings [7]. In visceral surgery, *E. coli* dominate, followed by Enterococcus species [8]. After lower abdominal tract surgery, the most frequent microorganisms isolated were *E. coli*, *Enterococcus* spp., *Streptococcus* spp., *P. aeruginosa*, and *S. aureus*. After upper abdominal tract procedures, the proportion of isolated staphylococci, *Klebsiella pneumoniae*, *Enterobacter* spp., *Acinetobacter* spp. and *Candida albicans* was higher, but that of *E. coli, Bacteroides fragilis* and *Clostridium* spp. was less [9]. In contrast, the members of the resident skin flora such as *S. aureus*, CoNS, *Cutibacterium acnes* and streptococci dominate in periprosthetic joint infections [10]. In vascular graft infections, staphylococci remain the most common bacteria [11]. The manifestation of SSI depends on various factors, such as site of surgery, age of the patient, underlying illness, amount and type of the microorganisms in the surrounding surgery field and their pathogenicity [12,13]. 

To eliminate the resident skin flora as the major source of SSI, the focus is on the following measures: pre-surgical antiseptic washing, if needed preoperative decolonization of *S. aureus*, deep skin antisepsis [14], antiseptic irrigation of the surgical field before suturing [15], glove change before inserting the sterile implant [16], biofilm reducing wound closure [17], antiseptic suture material [18], and perioperative parenteral antibiotic prophylaxis [19,20]. Due to the prevalence of SSI and the resulting high costs, every effort must be made to reduce the SSI rate. For example, the SSI rate in Germany in the period 2010–2016 was 4.9% with median case costs for the SSI group of €19,008 compared to €9040 for patients without SSI. The median underfunding of SSI was identified at €1534 per patient [21]. 

Skin antisepsis is a key element for the prevention of SSI. A promising starting point for the prevention of SSI is to improve the depth of preoperative skin antisepsis by reaching the hair follicles. One possibility could be the use of nanocarriers for an optimized delivery of antiseptics into the hair follicle. Thus, the efficacy of skin antisepsis could be increased and the intraoperative carryover of bacteria from the surgical field to the wound could be decreased. Nanoparticulate drug delivery systems show a significantly deeper follicular penetration than non-particulate substances when an external force like massage is applied [22]. Thereby, a movement of the hair shaft is induced which causes a mechanical interaction between the nanoparticles, the intrafollicular stratum corneum and the hair shaft, which together act as a kind of ratchet, also known as the ratchet effect [23]. Following the transport of the nanoparticles to the hair follicle, a release of the encapsulated drug can be induced by various trigger mechanisms [24]. Polymeric nanocapsules (NCs) containing *o*-nitrobenzyl groups are known for their fast photolysis under UV irradiation [25,26] since these compounds are known to have excellent photolytic properties [27] under mild irradiation conditions [28,29]. Recently, it was shown that low-dose ultraviolet A (UVA) radiation at about 365 nm is suitable for an intrafollicular release of a model drug from polyurethane NCs containing *o*-nitrobenzyl groups in the shell [26]. In the present study, biocompatible hydroxyl ethyl starch nanocapsules (HES-NCs) prepared by a miniemulsion process were utilized for this approach. HES is well suited for translation into the clinical routine due to its biodegradability and high biocompatibility which is comparable to native starch [30,31]. Furthermore, it is known for its presence of modifiable chemical groups as well as tailorability [32]. The material was equipped with photolytic characteristics by the incorporation of *o*-nitrobenzyl groups into the capsule shell, which enabled an efficient release of encapsulated ethanol (EtOH) as well as the model drug sulforhodamine 101 (SR101) after UVA irradiation. 

**Figure 1:**
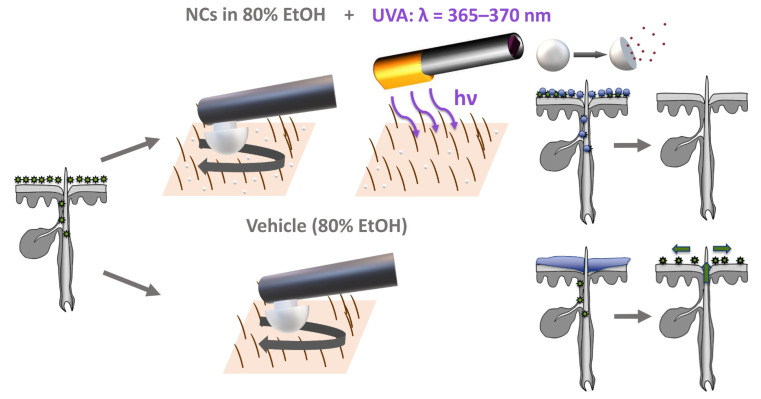
Application of the hydroxyethyl starch nanocapsules (HES-NCs) in 80% ethanol (EtOH) on colonized ex vivo porcine skin with a massage device and subsequent irradiation of the skin surface with a novel LED device emitting at a peak wavelength of 365–370 nm which leads to an intrafollicular release of EtOH from the HES-NCs resulting in a decolonization of the skin surface and the hair follicles. After application of the pure vehicle (80% EtOH) the skin surface is decolonized but the risk of a recolonization of the skin from deeper parts of the hair follicle remains.

Light emitting diodes (LEDs) are advantageous for biomedical applications [33] since they are compact, have stable output characteristics and can be mounted on various types of instruments. Furthermore they represent potential substitutes for mercury vapor lamps [34] and provide a narrow emission spectrum, which can be tailored to the application [26] accompanied by an increase in lifetime and reduced power consumption [35]. Therefore, we decided to use a novel LED-based device for the emission of UVA radiation at a peak wavelength of 365–370 nm as a trigger for the release of EtOH from the HES-NCs (Figure 1). In this context, we hypothesize that the HES-NCs, unlike the non-particulate vehicle, reach the deeper parts of the hair follicles and decontaminate them after UVA-triggered release of EtOH. In contrast, pure EtOH only reaches the skin surface and the upper infundibulum of the hair follicle [6], which is not sufficient for deeper decolonization. Based on this, it can be assumed that, especially in the case of prolonged surgical interventions, recolonization of the skin surface by endogenous microorganisms originating from the hair follicle can occur [5,14], which increases the risk of developing SSI. To accomplish the utilization of the newly synthesized HES-NCs in preoperative skin antisepsis, the aim of this work was the transfer to an antiseptic continuous phase (EtOH) as well as the physicochemical and toxicological characterization of the system. The physicochemical characterization was carried out using EtOH as well as phosphate-buffered saline (PBS) to assess the applicability of the system in aqueous phases. The toxicological characterization was performed using the outer phase PBS to exclude a bias due to EtOH-related cell toxic effects. We further applied the system on ex vivo porcine skin to evaluate follicular penetration as well as drug release characteristics. For the model visualization of the intrafollicular release of the model drug SR101, cyclohexane (CH) turned out to be the ideal outer phase, as its physicochemical properties help to indicate the localization of SR101. We finally elucidated the bactericidal properties of the HES-NCs in EtOH in combination with the novel UVA LED-based lamp using ex vivo porcine skin. 

## 2. Materials and Methods

### 2.1. Materials for HES-NC Synthesis

NXG (2-nitro-*p*-xylylene glycol) (TCI, Eschborn, Germany), toluene diisocyanate (TDI) and sulforhodamine 101 (SR101) (both Sigma-Aldrich, Steinheim, Germany), ethanol (EtOH) (GC grade, Merck KGaA, Darmstadt, Germany) and cyclohexane (CH) (HPLC grade, Acros Organics, Nidderau, Germany) were of commercial grade and used without further purification. Hydroxyethyl starch (HES) aqueous infusion solution (10%, Fresenius Kabi, Bad Homburg, Germany) contained 0.9% sodium chloride. Lubrizol (polyisobutylenesuccinimide pentamine, mw = 384–875 g·mol^−1^, determined from GPC, HLB (hydrophilic lipophilic balance) < 7, containing 50:50 wt% mineral oil as a diluent) (Lubrizol, Rouen en Seine Maritime, France) was used as a surfactant. Milli-Q water was used as aqueous phase throughout the experiments. Stock solutions of Lubrizol (1% in CH) and SR101 solutions (10^−3^ M in water and 5 × 10^−3^ M in EtOH-water, 1:1) were prepared and stored at room temperature protected from light.

### 2.2. Preparation of HES-NC Samples

NXG, HES solution, SR101 solution and EtOH were mixed together and stirred for 5 min to prepare the samples 1144b to g with the corresponding proportions as shown in Table 1. Then, a solution of Lubrizol in CH (7.5 g) was added. After stirring for 1 h with a magnetic stirrer for pre-emulsification, the miniemulsion was prepared by ultrasonication of the mixture for 120 s at 70% amplitude (Branson sonifier W450 Digital, tip size 12.5 mm, pulse 10 s, pause 20 s) under ice cooling. Then 85 mg of TDI dissolved in CH/Lubrizol (2 g) was added slowly to the miniemulsion within 5 min at room temperature. For the synthesis of the HES-NCs 1212, 12 small batches immediately after sonication were combined in one big flask and stirred at 700 rpm to obtain a higher formulation volume but still ensure an appropriate sonication process. To ensure that a reliable miniemulsion was obtained from the sonification, the volume and components of each batch for sample 1212 were identical to those for samples 1144. 1.02 g of TDI in 24 g of Lubrizol solution was added slowly within 10 min. The surface crosslinking was carried out upon stirring at 700 rpm for 24 h at 25 °C. To avoid solar irradiation, vessels were covered with aluminum foil. The obtained dispersions of HES-NCs in CH were used for characterization by dynamic light scattering (DLS) and transmission emission microscopy (TEM).

### 2.3. Characterization of the HES-NC Samples by Dynamic Light Scattering (DLS) and Transmission Electron Microscopy (TEM)

The size of the HES-NCs and their size distribution were measured by dynamic light scattering (DLS) (Zetasizer nano ZS, Malvern Instruments Ltd., Worcestershire, UK) at 20 °C under the scattering angle of 173° at a wavelength of 633 nm. DLS measurements give a z-average size (z-avg), which is intensity mean, and the polydispersity index (PDI), which provides information about the width of the particle size distribution. For the measurement, the CH dispersion was diluted 100 times. Particle sizes and PDIs are given as the average of 12 measurements of one representative aliquot. The size of the HES-NCs and their size distribution after transfer in EtOH or PBS with different surfactants were analyzed with four measurements and 12 runs per measurement. Data were analyzed based on their z-avg and their number as well as the PDI. The particle dispersion was diluted between 20 and 100 times before measurement.

The morphology of the HES-NC samples was studied using a transmission electron microscope (TEM) (JEM 1400, Jeol Ltd., Tokyo, Japan) operating at an accelerating voltage of 80 kV. A total of 10 μL of the original dispersion was diluted with 5 mL of CH, and then 3.0 μL of the diluted sample was placed on a 400-mesh carbon-coated copper grid and dried at room temperature overnight. In case of transfer into water or EtOH, the dispersions were diluted with correspondent solvents.

### 2.4. Transfer of HES-NCs to EtOH and PBS

The NC dispersion was diluted 1:5 in CH and centrifuged at 1400× *g* for 30 min at room temperature in 60 mL glass vials. The supernatant was removed, and the pellet was resuspended in EtOH (80%) or PBS (PeproTech EC Ltd., London, UK) without or with addition of surfactants in an ultrasonic bath with open cap for 30 min. The dispersion was again centrifuged and resuspended in fresh EtOH or PBS without or with addition of surfactants. Resuspension was again done in an ultrasonic bath. The following surfactants were used in 0.1 % (*w*/*v*) concentration: Tween^®^ 20 (Sigma Aldrich, St. Louis, MO, USA), betaine monohydrate (Sigma Aldrich, St. Louis, MO, USA), cetyltrimethylammonium chloride (CTMA-Cl, Sigma Aldrich, St. Louis, MO, USA), Nonidet^®^ P40 (NP40, AppliChem GmbH, Darmstadt, Germany).

### 2.5. Examination of In Vitro Release of the Model Drug SR101 and EtOH

SR101 was used as a model drug for testing the release after UVA-cleavage in CH and EtOH without and with addition of surfactants. The NC dispersion (1 mL) was diluted in 3 mL of the appropriate solvent. Then, 150 µL were transferred to a glass vial and irradiated with UVA radiation (peak wavelength of λ = 367 nm) for 1–7 min. The dispersion was placed directly onto an LED (NCSU033B, Nichia Corporation, Anan, Japan) (30 mW/cm^2^) or with 1.25 cm distance (6.8 mW/cm^2^) to the light source resulting in doses of 0.2–12.6 J/cm^2^.

Afterwards, 50 µL of the solution in CH was transferred to a black microtiter plate (96 well) and fluorescence was measured at 485_ex_/590_em_ nm after an exposure time of 0.5 s.

HES-NCs in EtOH were centrifuged (1400× *g*, 5 min) after irradiation and before measurement to separate non-cleaved fluorescent HES-NCs. The supernatants were used for fluorescence analysis. In PBS, the release of encapsulated EtOH was analyzed instead of SR101. The cleaved HES-NCs were removed by centrifugation and the EtOH concentration in the supernatant was determined by a Cer(IV)-based assay.

### 2.6. EtOH Detection

In 100 mL distilled water, 2 mL nitric acid (68%) was dissolved and 2.2 g Cer(IV)ammonium nitrate (Carl Roth GmbH + Co. KG, Karlsruhe, Germany) was added to reach a concentration of 0.04 M. Of this detection reagent, 100 µL were mixed with 100 µL of test solution and absorption was measured immediately at 415 nm. EtOH concentration was calculated by use of a calibration curve.

### 2.7. Cell Culture

The human cell line HaCaT (DKFZ, Heidelberg, Germany) was cultured in Dulbecco’s modified Eagle’s medium (DMEM, high glucose, PAN-Biotech GmbH, Aidenbach, Germany) supplemented with 10% (*v*/*v*) heat-inactivated fetal bovine serum (FBS) (Life Technologies, Carlsbad, CA, USA) and 2 mM L-glutamine (ccPro GmbH, Oberdorla, Germany) at 37 °C in a humidified atmosphere (37 °C, 5% CO_2_, Heracell™ 150i, Thermo Scientific, Waltham, MA, USA). The cells were subcultured twice a week with additional medium change weekly. For cell detaching, trypsin/EDTA (0.05%/0.02%, PAN-Biotech GmbH, Aidenbach, Germany) was used. Cell morphology was checked regularly. After detaching the cells, viability was assessed via trypan blue exclusion and cells were counted in a counting chamber (Neubauer improved).

### 2.8. Cytotoxicity Analysis

Cell viability was analyzed by a colorimetric assay using the yellow tetrazolium salt 3-(4,5-dimethylthiazolyl-2)-2,5-diphenyltetrazolium bromide (MTT) that is reduced by viable cells to a purple formazan. A HaCaT cell suspension with 0.1 × 10^6^ cells/mL was prepared and 100 µL of the suspension were added per well of a 96 well plate. After incubation for 48 h, the cells were exposed to the test compounds in decreasing concentration for 1 h and 3 h, respectively. Afterwards, cells were washed with PBS/HBSS (ccPro GmbH, Oberdorla, Germany) and 100 µL MTT solution (0.5 mg/mL) (Sigma-Aldrich, St. Louis, MO, USA) per well were added and incubation was continued for 3 h at 37 °C. The supernatant was decanted and for formazan solubilization, 100 µL MTT-elution solution (2-Propanol/HCl) (both Carl Roth GmbH + Co. KG, Karlsruhe, Germany) was added per well. The 96 well plate was incubated protected from light for 15 min while shaking. Absorbance was measured with a microplate reader (BioTek PowerWave XS, Agilent Technologies, Santa Clara, CA, USA) at 550 nm and 620 nm. Sodium dodecyl sulfate (SDS, 0.08%) (AppliChem GmbH, Darmstadt, Germany) served as a positive control. 

### 2.9. Examination of Ex Vivo Follicular Penetration of HES-NCs and Release of the Model Drug SR101

#### 2.9.1. Preparation of Skin Samples

Due to its suitability for investigating follicular penetration ex vivo, porcine skin was utilized for the drug delivery experiments [36,37,38,39]. The porcine ears were obtained from a local butcher. The age of donor pigs was sixth months at the date of slaughter. Porcine ears without any visible injuries were selected for further examinations and the experiments were executed not later than 48 h after slaughter to exclude possible post-mortem skin changes [40]. The porcine ears were cleaned under cold tap water and dried with paper towels and stored at 4 °C until the experiments [41]. The porcine ears were fixed on a polystyrene board covered by aluminum foil by using cannulas. In total, three test areas of 2 cm × 3 cm per ear (CH-based dispersion) or four areas of 2 cm × 3 cm (PBS-based dispersion) were selected and the included hairs were shortened without damaging the stratum corneum. The outer edges of the areas were covered by window color (fun & fancy, Marabu GmbH & Co. KG, Tamm, Germany) to avoid lateral spreading of the dispersions.

#### 2.9.2. Ex Vivo Application of HES-NCs

In total, *n* = 3 porcine ears were treated with the CH-based dispersion and *n* = 3 unrelated ears were treated with the PBS-based dispersion. The formulations were applied onto the test areas with a concentration of 20 µL/cm^2^ except one area per porcine ear which served as a negative control (untreated area). The porcine ears treated with PBS-based HES-NC dispersions further contained a test area with a solution of 0.001% (*w*/*v*) SR101 in PBS with 0.1% (*w*/*v*) betaine monohydrate as a control. After application, the test areas were manually massaged with a circular motion using a sonic wave device (NOVAVON pro, NOVAFON GmbH, Weinstadt, Germany) at a frequency of 4.2 Hz (250 BPM) for 2 min [42] followed by an incubation period of 5 min. Afterwards, one test area per ear containing a NC dispersion was irradiated by means of a UVA-LED module (OLM-018, OSA opto light GmbH, Berlin, Germany) from a distance of 3 cm (irradiance of 214.0 ± 2.3 mW/cm^2^, dose of 12.8 J/cm^2^, maximum emission wavelength of λ = 368.5 ± 1.5 nm) for 1 min. The corresponding application protocol is presented in Table 2. Subsequently, the skin areas were hardened by a cryospray (Solidofix^®^, Carl Roth GmbH + Co. KG, Karlsruhe, Germany) and skin biopsies of 0.5 × 0.5 cm were excised by means of a scalpel, transferred to cryotubes, and shock frozen with liquid nitrogen. The samples were stored at −20 °C until further preparation.

#### 2.9.3. Cryohistological Preparation and Confocal Laser Scanning Microscopy (CLSM)

The cryohistological preparation of skin biopsies was performed as described before [26]. Examination of the cryohistological sections was carried out via confocal laser scanning microscopy (CLSM) (LSM 700, Carl Zeiss AG, Oberkochen, Germany) on *n* = 9–10 hair follicles per group on each porcine ear using a laser emitting at 555 nm. The full procedure is likewise described in [26].

#### 2.9.4. Graphical Processing of CLSM Images

To quantify the intrafollicular UVA-dependent release of the model drug SR101 from the HES-NCs, we utilized an image analysis evaluating the mean brightness value of the fluorescence signal via ImageJ (Wayne Rasband, National Institutes of Health, Bethesda, MD, USA) as already described [26].

### 2.10. Development and Adaption of a UVA-LED Lamp for Microbial Reduction Experiments

**Figure 2:**
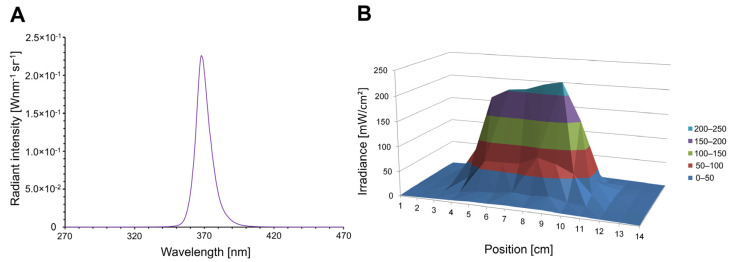
(**A**) Mean emission spectrum of the applied ultraviolet A (UVA)-LED. (**B**) Irradiance in the target range.

We developed a handheld, rechargeable battery-operated LED radiation source (LED lamp) for the ex vivo microbial reduction experiments to increase the translatability of the system for clinical application. The UVA-LED series OCL-490 applied in the LED lamp are specified with a peak wavelength of 365–370 nm, mean value 368.3 ± 0.5 nm. Further data are a full width at half maximum (FWHM) of 11.1 ± 0.3 nm, radiant intensity 3.1 ± 0.2 W/sr and a viewing angle (full angle) of 20°. The emission spectrum is shown in Figure 2A. All LED parameters were measured with a CAS-140 CT spectrometer (Instrument Systems, Munich, Germany) at a measurement current of 500 mA supplied by a National Instruments power source (Austin, TX, USA). The distribution of the irradiance on the skin has been measured by adjusting the LED lamp at a distance of 3 cm between skin and front window of the lamp, which is indicated in Figure 2B. We achieved an irradiance above 200 mW/cm² at an area of 12 cm². The UVA lamp contains six LEDs in total, a driver unit, a rechargeable battery pack, a passive heat sink and a sealed aluminum housing. Therefore, the lamp can be disinfected e.g., by wiping the housing surface with EtOH. To support the operator in adjusting the lamp position at 3 ± 1 cm distance above the skin, we integrated an optical distance measurement device and an optical indication of the actual distance at the back side of the LED lamp. We have determined the necessary measures to protect the operator (safety goggles) against UVA radiation. The irradiation dose of 13.1 J/cm² (Section 2.11.1) requested from the experimental set up is below the safety limit of 29 J/cm² according to 2006/25/EG Table 1.1, points a. und o. (safety regulations for non-coherent optical radiation) [43]. UVA irradiation as a trigger for intrafollicular release of antiseptics from NCs bears the advantage of having a potential antiseptic effect itself [44]. Furthermore, UVA light penetrates the dermis, which enables the targeting of hair follicles [45]. It is also able to trigger intrafollicular drug release at very low doses [26] from *o*-nitrobenzyl-based NCs without inducing skin heating, such as infrared A [46].

### 2.11. Analysis of Microbial Reduction

#### 2.11.1. Application of HES-NCs on Ex Vivo Porcine Skin and Sampling via Cup Scrub Technique

Porcine ears (*n* = 10), not refrigerated before the experiments and used at room temperature, were treated as described before (Section 2.9.1). One area served as a negative control and remained untreated. Another negative control was exposed to UVA radiation (irradiation time: 1 min, distance: 4 cm, irradiance: 218.7 ± 2.8 mW/cm^2^, dose: 13.1 J/cm^2^) using the LED device described in Section 2.10. (OSA Opto Light GmbH, Berlin, Germany). The other areas were treated with EtOH (80% including 0.1% betaine monohydrate, positive control), the HES-NC dispersion (in EtOH 80% with 0.1% betaine monohydrate), and with the HES-NC dispersion followed by UVA irradiation. 

The antiseptics were applied as follows: 20 µL/cm^2^ were administered to a skin area of 3 cm × 3 cm with a pipette and the skin was massaged for 2 min at 4.2 Hz (250 BPM) as described in Section 2.9.2. After air curing, an autoclaved steel ring (2 cm in diameter) was put on the skin and filled with 1 mL sterile inactivator solution TSHC (3% Tween^®^ 80 (AppliChem GmbH, Darmstadt, Germany), 3% saponin (Sigma Aldrich, St. Louis, MO, USA), 0.1% L-histidine (Carl Roth GmbH + Co. KG, Karlsruhe, Germany), 0.1% L-cysteine (Sigma Aldrich, St. Louis, MO, USA)). Then, the skin was rubbed with a sterile glass rod for 1 min to flood up the intrafollicular microbiome (cup scrub technique). This procedure was conducted on each area. 500 µL of the received suspension were added to 4.5 mL of inactivator solution and vortexed. Until use, samples were stored at 4–8 °C.

#### 2.11.2. Quantitative Determination of the Bacterial Skin Colonization

Samples were diluted, plated on Columbia blood agar (CBA) plates and incubated for 48 h at 37 °C. Afterwards, colony-forming units (CFU) were counted, and lg-reduction was calculated according to the following formula:

lg(reduction) = lg(CFU of control) − lg(CFU of treatment)(1)

#### 2.11.3. Identification of the Skin Microbiome by MALDI-TOF MS and Sanger Sequencing

Colonies differing in their phenotype (color, shape) were sub-cultured on CBA plates and identified using matrix assisted laser desorption ionization—time of flight mass spectrometry (MALDI-TOF MS) from fresh overnight sub-cultures.

Using a toothpick, colony biomass was transferred onto a disposable MALDI target plate (MBT Biotarget 96, Bruker Daltonics GmbH & Co. KG, Bremen, Germany) and 1 µL of 70% formic acid in HPLC grade water (both Honeywell GmbH, Seelze, Germany) was applied. A total of 1 µL bacterial test standard (BTS, Bruker Daltonics, Bremen, Germany) was added on a separate position. On both, the samples and the BTS, 1 µL α-cyano-4-hydroxycinnamic acid matrix (Bruker Daltonics GmbH & Co. KG, Bremen, Germany) was added after drying. The samples were analyzed with the MALDI Biotyper^®^ sirius instrument (Bruker Daltonics GmbH & Co. KG, Bremen, Germany) and the MBT Compass software version 4.1. Microorganisms consistently identified with a score value of ≥1.7 were assumed to be reliably identified. Microorganisms that could not be identified (score < 1.7) were subjected to the protein extraction method and re-analyzed. For this, colony material was resuspended in 300 µL HPLC grade water. Afterwards, 900 µL absolute EtOH (Carl Roth GmbH + Co. KG, Karlsruhe, Germany) was added. After two centrifugations (2 min, 14,800 rpm), the supernatant was discarded, and the pellet was dried for 5 min at room temperature followed by resuspension in 70% formic acid. To this suspension, 20 µL acetonitrile (Honeywell GmbH, Seelze, Germany) were added, the suspension was centrifuged again and 1 µL of the supernatant was applied to the MALDI targets. Afterwards, the procedure was carried out as described previously.

Microorganisms that could not be reliably identified by MALDI-TOF MS were identified using Sanger sequencing. DNA of the bacteria was isolated using the Genomic DNA/Nucleospin^®^ Microbial DNA kit (Macherey-Nagel GmbH, Düren, Germany) according to the manufacturer’s instructions. The DNA was stored at −20 °C until use. 16S rRNA were amplified with final concentration of 2 µM primer 27F (5′-AGA GTT TGA TCM TGG CTC AG-3′) and 16S-5 (5′-AAG GAG GTG ATC CAG CCG CA-3′) using Phire hotstart II DNA polymerase (Thermo Fisher Scientific, Waltham, MA, USA) kit. PCR was performed on a Biometra TRIO 48 thermocycler (Analytik Jena GmbH, Jena, Germany) using the following conditions: initial incubation at 98 °C for 30 s, 35 cycles of 98 °C for 5 s, 60 °C for 5 s, and 72 °C for 2 min, followed by a final incubation at 72 °C for 1 min. The amplicons were purified using a QIAquick Gel Extraction Kit (QIAGEN GmbH, Venlo, The Netherlands). DNA was sequenced according to the SANGER sequencing protocol by Eurofins Genomics Germany GmbH (Ebersberg, Germany) using the PCR primers. Finally, the forward and reverse sequences were aligned, cured and combined to 16S rRNA consensus sequences (Geneious, San Diego, CA, USA). The entire consensus 16S rRNA gene sequences were then subjected to BLAST analysis against the 16S rRNA (bacteria and archaea) of the NCBI nucleotide database [47]. The similarity scores (sc) were regarded as identifications of either the species level, if sc > 99% or at least the genus level sc > 97%. For an additional control, all isolates were subjected to Gram-staining and the microscopic images were compared with the respective results of the 16S rRNA identifications.

### 2.12. Statistical Analysis

Mean value comparisons were carried out via IBM SPSS^®^ Statistics 28 (IBM, Armonk, NY, USA). Normal distribution of the data was proven by the Shapiro–Wilk test. A Mann–Whitney U test (follicular penetration depth and fluorescence intensity) or a one-tailed Student’s *t*-test for paired samples (release of EtOH from the HES-NCs) was applied in case of two groups and a Kruskal–Wallis analysis of variance (ANOVA), followed by Bonferroni post hoc tests (DLS after phase transfer, cytotoxicity, follicular penetration depth), was applied in case of more than two groups. A one-way ANOVA with Tukey’s multiple comparisons was carried out for the microbial reduction data. All tests were carried out affording a significance of *p* < 0.05.

## 3. Results and Discussion

### 3.1. Synthesis and Physicochemical Characterization

**Figure 3:**
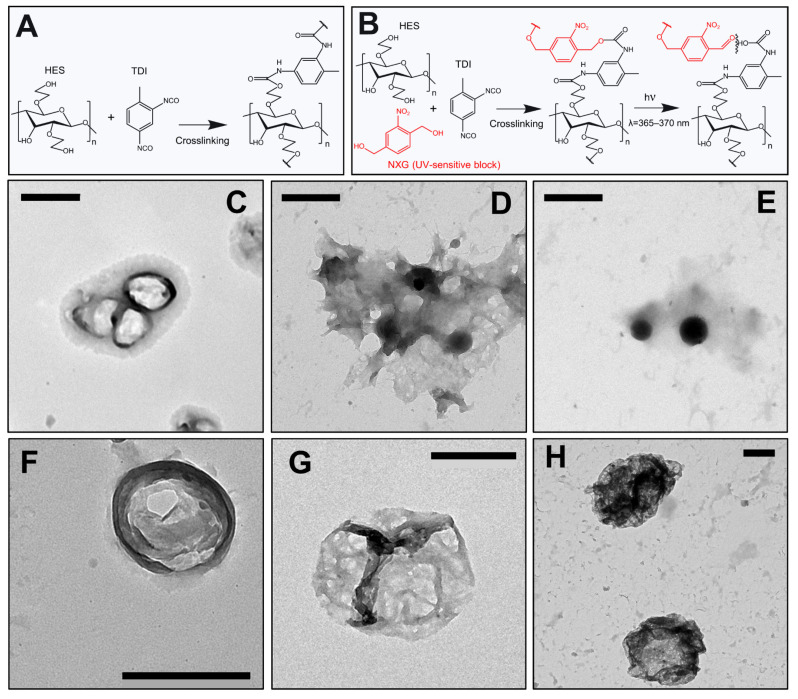
(**A**) Crosslinking of the HES chains with usual crosslinker. (**B**) Crosslinking of the HES chains with photocleavable co-crosslinker, and the UVA light induced cleavage of the cross bonds. Transmission electron microscopy (TEM) images of the formulations 1144d (**C**), 1144d in EtOH/water (**D**), 1144d in EtOH + 1%Tween 20 (**E**), 1144g (**F**), 1144g + UVA (**G**), 1212 (**H**). Scale bars correspond to 500 nm.

In this work, we used biocompatible HES as a shell forming polymer. In contrast to polyurethane, HES is a water-soluble polymer. In order to make it insoluble, crosslinking of hydroxyl groups was used, producing a water-insoluble 3D network. TDI was used as a main crosslinker, whereas photosensitive NXG was used as a co-crosslinker. When TDI acts as a sole crosslinker, non-cleavable bonds are formed (Figure 3A). By using both crosslinkers, *o*-nitrobenzyl blocks in the cross bonds make the shell photosensitive. Irradiation with UVA light breaks the cross bonds formed by the NXG crosslinker (Figure 3B) which induces disintegration of the capsules accompanied by the release of the content into the surrounding medium. 

In the framework of the synthesis, photoresponsive HES-NCs with a spherical shape were formed (Figure 3C,H) showing agglomerations as well as morphological alterations after the transfer to 80% EtOH (Figure 3D) which vanished after the addition of the surfactant Tween 20 (5%, Figure 3E). UVA radiation induced breakage of the capsule shell (Figure 3F,G). Since there are two types of cross-links, photocleavable as well as non-photo-cleavable, the capsule shell may not be completely destroyed. However, large openings with a high permeability for the encapsulated drug were formed. This effect of disintegration was already observed in polyurethane-based NCs containing a NXG component in the shell [26]. 

From the literature various starch-based NCs are known involving the possibility of a controlled drug release triggered by pH [48,49], GSH [50], glucose or temperature [51]. To our knowledge, the presented NCs are the first UVA-responsive *o*-nitrobenzyl-based HES-NCs.

### 3.2. Physicochemical HES-NC Characteristics after Transfer of the HES-NCs to PBS and EtOH

In order to further evaluate the optimal parameters for stabilization, we used DLS to investigate formulations after transfer to PBS and EtOH. Various surfactants were added to the respective phase in cytocompatible concentrations since the formation of adlayers to NC dispersions is a known phenomenon resulting in stabilization [52]. 

**Figure 4:**
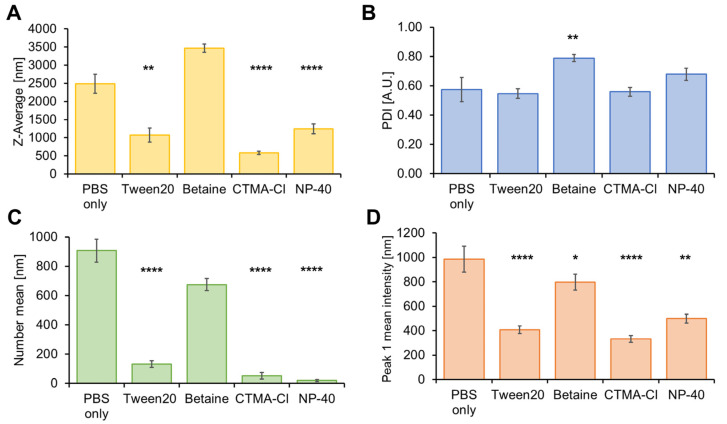
Dynamic light scattering (DLS) parameters (**A**) z-average, (**B**) polydispersity index (PDI), (**C**) number mean, (**D**) peak 1 mean intensity of the HES-NCs 1144d and g after transfer into phosphate-buffered saline (PBS). Different stabilization approaches by adding 0.1% (*w*/*v*) of the surfactants Tween 20, betaine monohydrate, CTMA-Cl or NP-40 are shown in comparison to PBS only. Significances in comparison to PBS only are indicated by asterisks with * = *p* < 0.05, ** = *p* < 0.01, **** = *p* < 0.0001 as determined by Bonferroni-adjusted post hoc tests after Kruskal–Wallis one-way analysis of variance (ANOVA). Mean values ± standard errors of the means (SEMs) of *n* = 4 to 30 samples with *n* = 3 to 4 measurements and 12 sub-runs each are presented.

In these experiments the HES-NCs 1144d-g were included. Next to the z-average (Figure 4A), the PDI (Figure 4B), the number mean (Figure 4C), which is the average size based on the number of the particles, and the mean intensity of peak 1 (Figure 4D), which is the proportionally largest peak of the particles’ intensity distribution, were used for assessing the quality of the transfer. Since a particle size of 600–800 nm is optimal for penetration into the hair follicle [53], z-average and number mean should be in this range. A comparable peak 1 mean (intensity) and small PDI (<0.7) are further criteria. When using PBS without surfactants, z-average, number mean and peak 1 mean were high. Data for HES-NCs in PBS with 0.1% betaine monohydrate are comparable. Nevertheless, number mean and peak 1 mean were in the desired range for follicular penetration using 0.1% betaine monohydrate. Utilization of Tween 20 (0.1%), CTMA-Cl (0.1%), and NP-40 (0.1%) led to a z-average and peak 1 mean intensity in the desired range but very low number peaks, meaning intensity of large particles overlaps the intensity of few small particles which could have been the result of dissolution.

**Figure 5:**
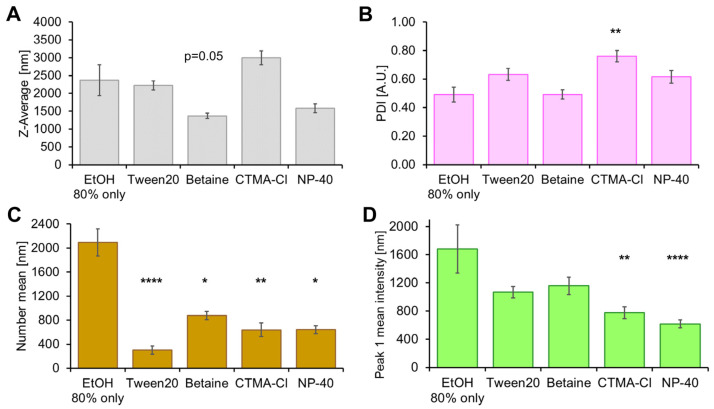
DLS parameters (**A**) z-average, (**B**) PDI, (**C**) number mean, (**D**) peak 1 mean intensity of the HES-NCs 1144d and g after transfer into EtOH (80%). Different stabilization approaches by adding 0.1% (*w*/*v*) of the surfactants Tween 20, betaine monohydrate, CTMA-Cl or NP-40 are shown in comparison to EtOH 80% only. Significances in comparison to EtOH only are indicated by asterisks with * = *p* < 0.05, ** = *p* < 0.01, **** = *p* < 0.0001 as determined by Bonferroni-adjusted post hoc tests after Kruskal–Wallis ANOVA. Mean values ± SEMs of *n* = 5 to 18 samples with *n* = 3 to 4 measurements and 12 sub-runs each are presented.

When transferring the HES-NCs to EtOH, data are most consistent for solutions containing betaine monohydrate (0.1%) (Figure 5A–D). Pure EtOH (80%), EtOH with Tween 20 and CTMA-Cl yielded high z-averages, while NP-40 resulted in a higher amount of small particles.

### 3.3. Cleavage of HES-NCs in CH, EtOH (80%) and PBS

**Figure 6:**
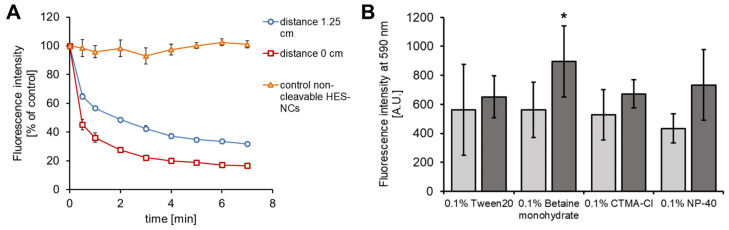
In (**A**) the relative fluorescence intensity of the model drug sulforhodamine 101 (SR101) to a non-irradiated control after irradiation of the HES-NCs 1144d with UVA from a distance of 1.25 cm (6.8 mW/cm^2^, blue, *n* = 6), 0 cm (30 mW/cm^2^, red, *n* = 8) and after irradiation of the non-cleavable HES-NCs 1144b (30 mW/cm^2^, orange, *n* = 4) is presented. (**B**) Release of SR101 as measured by fluorescence intensity of the supernatants (590 nm) after excitation at 485 nm without (light grey) and with cleavage of the HES-NCs (dark grey) showing a significant increase after irradiation of the HES-NC dispersion stabilized by 0.1% betaine monohydrate in EtOH (* = *p* < 0.05) as determined by one-tailed Student’s *t*-test for paired samples (*n* = 3 to 4 samples). Presentation of mean values ± SEMs in both graphics.

When irradiated with 365–370 nm, non-cleavable HES-NCs did not release SR101, which was indicated by a non-depleting fluorescence intensity (Figure 6A). When using cleavable HES-NCs, fluorescence intensity decreased as a result of cleavage accompanied with the release of SR101 to CH. The inverted release indication of the model drug SR101 is based on a recrystallization in apolar CH. A photobleaching effect of SR101 can be excluded in this context as it was shown in a previous study [26]. Cleavage efficiency depends on the distance to the light source and thus on the dose that is applied. Maximum cleavage was reached after 3 min irradiation with 30 mW/cm^2^ corresponding to a signal loss of approximately 80%. 

In our previous work involving *o*-nitrobenzyl-based polyurethane NCs we could reach a release of approximately 60% after 2 min by irradiation with 12 mW/cm^2^ [26]. Similar values were also obtained in other studies involving *o*-nitrobenzyl-based systems [54,55] which is in a comparable range to the HES-NCs utilized in this work. Compared to a micellar *o*-nitrobenzyl-based system presented by Jiang et al. [28] the release rate of the HES-NCs is similar when using approximately 20% of the irradiance. Although many UVA-responsive *o*-nitrobenzyl-based NCs have been published in previous papers [25,26,54,55], UVA-responsive *o*-nitrobenzyl-based HES-NCs are not known from the literature. Nevertheless, spyropyrane based UV-responsive starch nanogels were published before [56].

In EtOH, the highest fluorescence intensity was measured in the supernatant when HES-NCs with 0.1% betaine monohydrate were irradiated (Figure 6B). Here, a fluorescence increase of 59% (*p* < 0.05) was observed in the supernatant. Therefore, we decided to utilize betaine monohydrate for the HES-NC dispersions in the follow-up experiments.

In PBS, the fluorescence could not be measured, therefore the EtOH concentration was analyzed in the supernatants. The limit of detection (LOD) was calculated to be 0.10% EtOH, whereas the limit of quantification (LOQ) was about 0.37% EtOH. After cleavage of the HES-NCs in PBS, an EtOH concentration of 0.12–0.32% was measured in the supernatant. In comparison, in HES-NC samples without irradiation, no EtOH could be detected.

### 3.4. Cytotoxicity of HES-NCs

**Figure 7:**
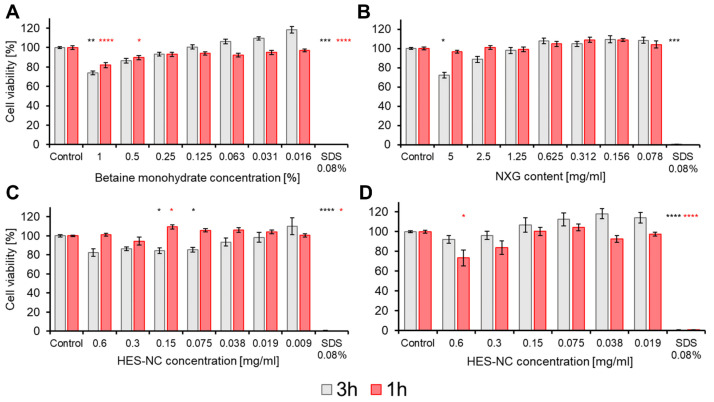
Cell viability as determined by 3-(4,5-dimethylthiazolyl-2)-2,5-diphenyltetrazolium bromide (MTT) assay for (**A**) betaine monohydrate, (**B**) NXG, (**C**) HES-NCs, (**D**) HES-NCs + UVA after 3 h (grey) or 1 h (red) of exposure. Significances in comparison to the control are indicated by asterisks with * = *p* < 0.05, ** = *p* < 0.01, *** = *p* < 0.001, **** = *p* < 0.0001 as determined by Bonferroni-adjusted post hoc tests after Kruskal–Wallis ANOVA. Mean values ± SEMs of *n* = 6 to 21 samples are presented.

Neither NXG used as the light sensitive linker in HES-NC production (Figure 7A), nor betaine monohydrate (Figure 7B) used as surfactant when transferring the HES-NCs to EtOH or PBS show any cytotoxicity towards HaCaT cells for contact times of 1 and 3 h in the applied concentrations since cell viability in comparison to the control was never <70%. Referring to the calculated IC_30_, the concentrations leading to cytotoxicity, are 7.1% and 1.5% for betaine monohydrate and 6.3 mg/mL for NXG within 3 h. Neither non-cleaved HES-NCs (Figure 7C), nor UVA-cleaved HES-NCs led to a cytotoxic reaction within 3 h. The high biocompatibility of HES-NCs is associated with previous works on human skin fibroblasts and immune cells [57,58,59].

### 3.5. Ex Vivo Follicular Penetration and Intrafollicular Release of SR101

**Figure 8:**
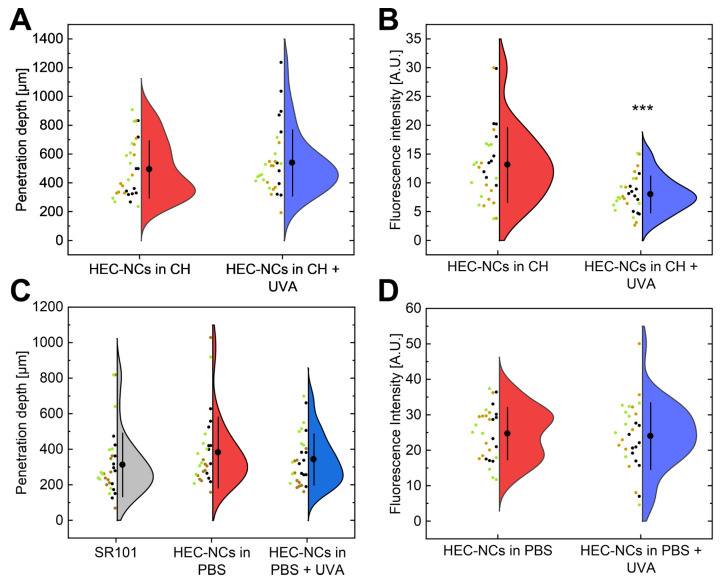
Violin plots, which show the distribution of individual data points including mean values ± standard deviations of *n* = 29 to 30 hair follicles of *n* = 3 donors. The three different colors of the data points serve to assign the hair follicles to the respective donor. An average follicular penetration depth of 499 µm could be reached by HEC-NCs in cyclohexane (CH) (1144f) (**A**) as well as an intrafollicular release of the model drug SR101 by 40% as indicated by loss of fluorescence intensity (**B**). After transfer of the HEC-NCs into PBS an average follicular penetration depth of 383 µm was observed (**C**) which was not significantly altered after irradiation with UVA but was slightly increased in comparison to a pure solution of the model drug SR101 in PBS (314 µm). No change in fluorescence intensity was detectable using PBS as continuous phase (**D**). Significances are indicated by asterisks with: *** = *p* < 0.001 (Mann–Whitney U test).

After application of the HES-NCs in CH and subsequent evaluation of cryohistological sections by CLSM, a mean follicular penetration depth of 499 ± 199 µm (mean value ± standard deviation) (without UVA) and 541 ± 230 µm (with UVA) was observed (Figure 8A). Indicated by a 40% loss of mean intrafollicular fluorescence intensity after irradiation with UVA, a release of the model drug SR101 from the HES-NC was detected (*p* < 0.001) (Figure 8B). 

**Figure 9:**
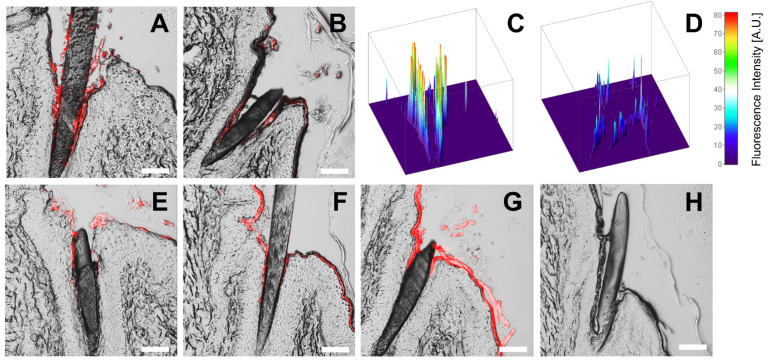
Representative confocal laser scanning microscopy (CLSM) images showed a significant difference of fluorescence intensity (depicted in red) before irradiation of HES-NCs in CH (1144f) (**A**) and after irradiation with UVA light (**B**) which indicated a release of the model drug SR101 from the HES-NCs (by the decrease in fluorescence intensity). The intrafollicular fluorescence intensity is further shown in pseudo-color 3D-plots for HES-NCs in CH before irradiation in (**C**) and after irradiation with UVA light in (**D**). No difference in fluorescence intensity and a slightly reduced penetration depth was noticed after transfer of the HES-NCs to PBS before (**E**) and after (**F**) irradiation with UVA but still higher in comparison to pure SR101 solution (**G**). No fluorescence was observed in untreated follicles (**H**). Scale bars correspond to 100 µm.

Representative CLSM images as well as corresponding false-color plots presenting the fluorescence intensity as an indicator for the release state of SR101 are depicted in Figure 9A–D. After transfer of the HES-NC from CH to PBS the size of the HES-NCs increased due to aggregation as already presented in Figure 4. Due to the increase in the mean diameter of the particles, a lower penetration depth of 383 ± 199 µm (without UVA) and 344 ± 144 µm (with UVA) was observed. However, the follicular penetration depth was 22% higher compared to the pure SR101 solution in PBS (314 ± 179 µm) (Figure 8C). Furthermore, the measured fluorescence intensity showed no differences before and after irradiation with UVA (Figure 8D).

Numerous previous studies show the possibility of penetration of spherical nanoparticles into hair follicles inducing an enhanced follicular transport of therapeutics [60,61,62,63,64,65,66,67,68,69,70] and subsequent triggering of release by various external as well as internal trigger mechanisms. Several physical trigger mechanisms, based on diffusion [71] or infrared light [46] as well as chemical trigger mechanisms based on pH [72,73] or proteolysis [74,75] were demonstrated in previous studies. In a recent work, we have already demonstrated an intrafollicular release of the model drug SR101 from UVA-responsive NCs [26]. The capsules used were made of polyurethane and contained the photoresponsive group NXG, which was also used in the present study. Using these particles with an approximate size of ≈700 nm, a follicular penetration depth of about 500 µm as well as an intrafollicular release of the model drug were demonstrated. These data compare well with the penetration depth and release of the HES-NCs in CH used in the present work (Figure 8A,B). 

After transfer of the HES-NCs into PBS, an increase in the z-average and PDI of the particles was observed corresponding to a decrease in follicular penetration depth. It is known that particles with an average diameter of 600 nm are best suited for follicular penetration [42,53] due to the so-called ratchet effect, which requires the particle size to match the space between the cuticle and the intrafollicular stratum corneum [23]. Because of an increase in the mean particle size (Figure 4) this effect did not occur for a certain proportion of the particles, as they were trapped in the described interstitial space. In our previous study, we postulated that the decrease in fluorescence intensity is associated with the release of the model drug SR101 from the polar intraparticulate environment into the apolar extraparticulate environment [26]. Since a polar outer phase was used here, SR101 did not show any measurable alterations in fluorescence intensity. Therefore, the water-based system was not suitable for an indication of release. 

### 3.6. Ex Vivo Microbial Reduction

**Figure 10:**
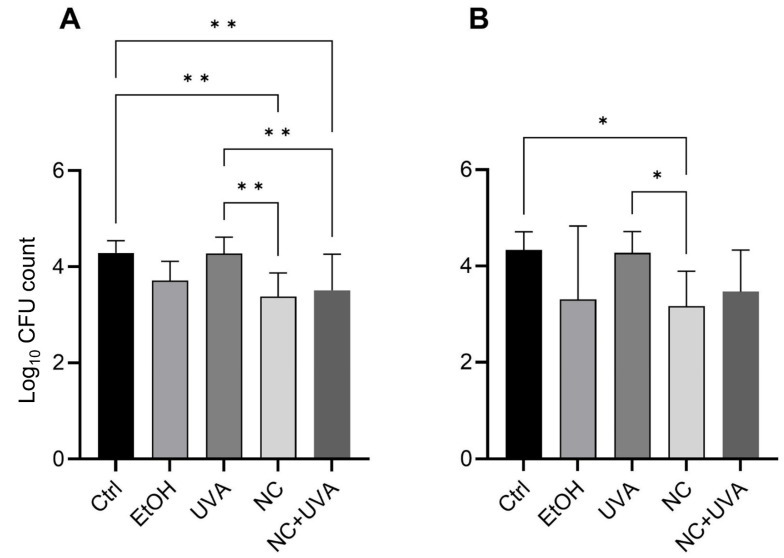
Log_10_ values of ex vivo porcine skin colonization for untreated skin (Ctrl) after treatment with 80% EtOH with betaine monohydrate (EtOH), UVA irradiation (UVA), HES-NCs in 80% EtOH with 0.1% betaine monohydrate (NC) and HES-NCs in 80% EtOH with 0.1% betaine monohydrate + irradiation with UVA (NC + UVA) for bacteria cultivated under aerobic conditions (**A**) and bacteria cultivated under anaerobic conditions (**B**). Significances between the treatments are indicated by asterisks with * = *p* < 0.05, ** = *p* < 0.01 as determined by Tukey post hoc tests after one-way ANOVA. Mean values ± standard deviations are presented for *n* = 10 porcine ear skin samples.

Treating porcine skin with EtOH (80%) reduced the microbial load by 0.75 ± 0.51 lg levels (aerobic conditions) and 1.27 ± 1.46 lg levels (anaerobic conditions) whereas treatment with HES-NCs in EtOH with betaine monohydrate led to a reduction of 1.08 ± 0.55 and 1.36 ± 0.63 lg levels, respectively. Pure EtOH (80%) had minor effects that were not statistically significant. When the HES-NCs were cleaved after application, reduction was about 0.95 ± 0.93 and 1.05 ± 0.83 lg levels meaning that UVA-induced cleavage of the HES-NCs induced no enhanced microbial reduction. The number of CFU of bacteria cultivated under aerobic conditions was statistically significantly reduced by the HES-NC dispersion and the UVA-cleaved HES-NCs in comparison to UVA-treated and the control porcine skin (*p* < 0.01, Figure 10A). HES-NCs led to a statistically significant reduction in colonization with bacteria cultivated under anaerobic conditions in comparison to the control and to UVA treated skin (*p* < 0.05, Figure 10B). An antiseptic effect caused by irradiation with UVA light as reported in previous studies [44] could not be observed.

Compared to human skin, porcine skin has a concordance of around 97% in the genera found with a higher colonization of 2 log levels [1]. Staphylococci and *Corynebacterium* ssp. were mainly found on porcine skin under aerobic culture conditions on the *n* = 10 porcine ears conducted in this study. Besides the *Propionibacteriaceae*, these also make up the main part of the colonization of human skin [2,76]. On control areas they made up the main proportion with 54.1 ± 25.0% and 33.1 ± 22.5%, respectively. On EtOH-treated skin areas, proportions were similar (57.6 ± 36.3% and 30.0 ± 27.5%). The proportion of *Curtobacterium* ssp. was rising (6.9 ± 1.5% to 56.6 ± 9.3%). Interestingly, on skin areas treated with NC and UVA, 72.0 ± 30.8% of the bacteria were staphylococci, whereas 27.5 ± 22.4% were *Corynebacterium* ssp. In contrast, only in one sample, curtobacteria (18.8%) were found. Rising proportions of staphylococci indicate a stronger reduction in further microorganisms with lower abundance as of the genera *Dietzia, Gordonia, Levilactobacillus, Rhodococcus, Micrococcus, Bacillus, Pseudomonas* and others. In most samples, mainly staphylococci and *Corynebacterium* ssp. were found after treatment with EtOH or NC + UVA. Under anaerobic conditions, proportions were similar. Staphylococci accounted for 62.1 ± 22.9% in control samples, 71.1 ± 32.7% on EtOH-treated samples, and 84.7 ± 15.5% in NC + UVA treated samples. The proportion of *Corynebacterium* ssp. was steady (31.1 ± 27.7%, 30.0 ± 29.5%, 27.2 ± 11.3%).

The results provide a first indication of an influence on the composition of the skin microbiome possibly resulting from differing proportions of species in the samples. Whether the HES formulation actually has a significant effect should nevertheless be evaluated in the context of a larger in vivo study.

Besides nanoparticle-free approaches for the hygienic treatment of hair follicles, such as the binding of sebum via triethanolamine [77,78], it was shown in several previous works that the delivery of antiseptics to hair follicles by using NCs is possible [79] and induces enhanced skin decolonization [80,81,82,83]. Since the enhanced antiseptic effect of the HES-NCs was not statistically significant in this experiment, we conclude that the hypothesized reduced recolonization should be elucidated in future experiments under in vivo conditions, where sebum flow from the hair follicles is present.

Based on the International Commission on Non-Ionizing Radiation Protection (ICNIRP), the applied dose of 219 mW/cm^2^ (Section 2.11.1) corresponds to 48% of the threshold for actinic damage and 36% of the threshold for one minimal erythema dose. According to Lohan et al. [84] this dose is still below the threshold (0.5 MED) causing irreversible lipid oxygen species in skin. Thus, the applied doses of UVA in the microbial reduction experiment can be considered as safe.

## 4. Conclusions

In the present study we were able to successfully produce photoresponsive HES-NCs in 80% EtOH by incorporating an *o*-nitrobenzyl derivative. Furthermore, we showed that UVA-induced photocleavage as well as efficient release of a model drug EtOH is possible with this system which could be utilized for the delivery and release of numerous hydrophilic drugs. It was found that by using the surfactant betaine monohydrate, the HES-NCs exhibited the highest stability in the target medium water/EtOH. Furthermore, the use of HES as a basic agent for NC manufacture resulted in a cytocompatible product. By using the novel HES-NCs, we achieved deep follicular penetration and intrafollicular release of the model drug EtOH using CH as a continuous phase. Nevertheless, we showed that detection of release of the model drug in the hair follicle is difficult using polar continuous phases. The present work clearly demonstrated the importance of HES-NC stability for follicular penetration. The latter was limited after transfer of the HES-NCs to PBS because agglomeration occurred and the HES-NCs were thus outside the size range suitable for follicular penetration. For the ex vivo microbial reduction experiments we successfully developed a powerful LED device to increase the translatability of the system. Regardless of the reduced penetration depth due to agglomeration, HES-NCs in EtOH led to a significant reduction in bacteria ex vivo. EtOH without HES-NCs had minor effects which, however, were not statistically significant. These data provide first indications that the application of the newly developed HES-NCs in combination with UVA light is conceivable for advanced skin antisepsis. Nevertheless, the hypothesis of reduced recolonization when using NCs should be elucidated in future experiments under in vivo conditions. In conclusion, when synthesizing the capsules, the targeted application area should be considered in order to avoid possible incompatibilities.

## Figures and Tables

**Table 1:** Composition of the dispersed phase of the HES-NC samples and their DLS characterization (cyclohexane = CH, ethanol = EtOH, hair follicle = HF, hydroxethyl starch = HES, z-average = z-avg, polydispersity index = PDI, phosphate-buffered saline = PBS, 3-(4,5-dimethylthiazolyl-2)-2,5-diphenyltetrazolium bromide = MTT, 2-nitro-*p*-xylylene glycol = NXG, sulforhodamine 101 = SR101).

Sample	Experiment	NXG [mg]	SR101 Aqueous Solution [mg]	EtOH [mg]	H_2_O [mg]	HES 10% [mg]	z-Avg [nm] in CH	PDI in CH
1144b	Release in CH (non-cleavable control, Section 3.3)	-	200	300	-	500	366	0.055
1144d	Phase transfer, release in CH and EtOH (Section 3.2 and Section 3.3)	50	200	300	-	450	356	0.083
1144e	HF penetration in PBS (Section 3.5)	50	200 *	200	100	450	432	0.127
1144f	HF penetration in CH (Section 3.5)	50	100 *	250	50	450	435	0.187
1144g	Phase transfer (Section 3.2)	50	-	450	-	500	503	0.144
1212	MTT assay, microbial reduction (Section 3.4 and Section 3.6)	50	-	450	-	450	613	0.215

* 5 × 10^−3^ M solution of SR101 in EtOH-water (1:1) was used.

**Table 2:** Treatment scheme of ex vivo porcine skin with HES-NC dispersions in CH and PBS with and without UVA irradiation.

Experiment	Area	Treatment
CH-based HES-NC dispersion (*n* = 3)	A	Negative control (untreated)
	B	HES-NC
	C	HES-NC + UVA
PBS-based HES-NC dispersion (*n* = 3)	A	Negative control (untreated)
	B	SR101 solution
	C	HES-NC
	D	HES-NC + UVA

## Data Availability

The data are available from the corresponding author upon reasonable request.
